# A Novel Bioengineering Approach to Breast Cancer Invasion In Vitro Through Enhancing the Value of Existing Breast Cancer Epithelial Lineages

**DOI:** 10.3390/cells15201882

**Published:** 2026-10-15

**Authors:** Ethan Webb, Kirsty Goncalves, Stefan Przyborski

**Affiliations:** 1 Department of Biosciences, Durham University, Durham DH1 3LE, UK; 2 Reprocell Europe Ltd., Glasgow G2 4SQ, UK

**Keywords:** cancer-associated fibroblasts, tissue bioengineering, extracellular matrix, breast cancer, migration, invasion, in vitro

## Abstract

Breast cancer incidence is rising globally. The emerging literature implicates cancer cell interactions with their surrounding microenvironment as a clinically significant driver of cell migration and metastasis. Current in vitro modelling approaches often rely on basic two-dimensional culture practices, a microenvironment far removed from the native tissue. This simplicity fails to recapitulate key aspects of the tumour environment that are critical to disease progression. This study has built upon three-dimensional culture technology to describe a breast cancer invasion assay with enhanced complexity compared with standard approaches. Three key breast epithelial cell lines, namely MCF-10A, MCF-7 and MDA-MB-231, representative of increasing invasiveness, were applied to in vitro systems of varying complexity. Through enhancing the geometry of the culture system, and inclusion of stromal populations, we have successfully improved breast cancer invasion modelling in vitro, exemplified by a demonstrated response to a test compound. The incorporation of a tissue-specific stromal foundation, upon which each epithelial cell lines were cultured, allowed for interaction between neosynthesised extracellular matrix and stromal and epithelial cells, whilst providing a dense physical network for cancer cells to migrate through. This has introduced several microenvironmental factors consistent with the native tissue but lacking in other more simplistic culture systems and has enhanced the value of commonplace cancer cell lines. This novel bioengineered culture system also lends itself to in-depth fundamental mechanistic insights into aspects of cancer cell biology, including interactions with the tissue environment and the role of cancer-associated fibroblasts in disease progression.

## 1. Introduction

Breast cancer is one of the most diagnosed cancers globally, accounting for up to 10.4% of all cancer diagnoses [1,2,3], and is the leading cause of death in women worldwide. In 2020 alone, 2.3 million new cases were diagnosed worldwide and 684,996 deaths were recorded [4]. This demonstrates the significant burden breast cancer poses to healthcare systems, and higher mortality rates in developing countries highlight substantial disparity in access to treatment. Given the burden of breast cancer on a global scale, there is a need for a more reliable system capable of modelling aspects of the disease at the cellular level to provide novel mechanistic insights and reliable pre-clinical predictive screening.

The highly heterogenous nature of breast cancer complicates both its treatment and our understanding of the underlying pathological mechanism [5]. Early analysis of breast cancer tumours divides them into four broad subtypes based on their localisation: lobular carcinoma in situ (LCIS), lobular invasive carcinoma (LIC), ductal carcinoma in situ (DCIS) and invasive ductal carcinoma (IDC) [6]. Further divisions are made based on expression of hormone receptors (oestrogen (ER+) and progesterone (PR+)), proliferation markers or epidermal growth factor receptor 2 (HER2) [1,2,6,7,8,9]. Expression of such factors plays integral roles in the pathological mechanism of each breast cancer subtype, impacting clinical presentation, prognosis and treatment options.

The invasion potential of cancer cells is a crucial factor that contributes to metastasis and treatment outcome. Although all breast cancer subtypes have the potential to metastasise, there is a clear correlation between rate and location of tumour spread among subtypes. Cancers that do not express hormone receptors (triple-negative (TNBC)) and those that are HER2+ have a higher rate of metastasis compared with hormone receptor-positive cancers [3,10,11,12]. This differing propensity to migrate is thought to be driven by complex microenvironmental interactions. Specifically, physical signalling between tumour cells and their environment is known to drive changes in gene, transcriptomic and metabolic profiles, leading to adoption of migratory phenotypes [13,14,15,16,17]. Cancer-associated fibroblasts (CAFs) develop in response to microenvironmental remodelling and play a role in the adoption of a migratory phenotype by tumour cells through release of transforming growth factor-β (TGFβ), which, via Smad activation, modulates gene transcription and acquisition of a mesenchymal phenotype [18,19,20,21,22,23,24,25,26,27]. Together these mechanisms suggest that both biochemical and physical interactions between cancer cells and their microenvironment, along with intrinsic cellular properties, contribute to the overall invasive phenotype of breast cancer subtypes.

Due to the complex cellular and molecular interactions between tumour cells and their microenvironment, combined with the heterogeneity of breast cancer in vivo, reliable in vitro modelling of the diseases is challenging. Current experimental methods either utilise traditional two-dimensional (2D) culture practices, which lack physiological relevance and complexity [28,29,30,31], or rely on three-dimensional (3D) hydrogel or organoid systems, both of which are associated with limitations. Although the use of hydrogels as a 3D matrix for breast cancers is widely described in the literature, the use of animal-derived exogenous extracellular matrix (ECM) introduces variability through limited or undefined composition and inter-species compatibility considerations [32,33,34,35]. Organoids overcome some of the disadvantages associated with hydrogel use, as they are self-organising 3D structures that do not require addition of exogenous components and can be scaled up for high-throughput drug discovery [32,36,37,38,39]. However, the use of organoids is also associated with shortcomings, as spheroid cultures rarely recapitulate native tissue structural anatomy, which, combined with restricted reproducibility and limited nutrient diffusion distance, restricts their potential to investigate migratory potential.

Accordingly, there is demand for a more advanced in vitro model that recapitulates the complex nature of breast cancer and the interactions of cancer cells with their surrounding microenvironment. Previously, we have described the use of Alvetex^®^, a porous polystyrene scaffold, as the foundation upon which a range of epithelial tissues (for example, skin [40,41,42,43], airway mucosa [44], intestine [45,46,47]) have been bioengineered. The defining feature of these models is the ability of a stromal fibroblast population to neosynthesise endogenous, tissue-specific ECM capable of supporting an intact epithelium and accompanying basement membrane [43,44]. In this study, we apply this technology to generate a more physiologically relevant 3D invasion model capable of both screening migratory potential and enhancing the predictive value of existing breast cancer lines through the introduction of stromal–epithelial interaction. This novel in vitro bioengineered assay system lends itself as a valuable research tool for a myriad of possible applications including drug screening, personalised medicine, and the study of fundamental cell and molecular mechanisms that underpin breast cancer progression.

## 2. Materials and Methods

### 2.1. Maintenance of Breast Epithelial Cell Lines

The MCF-7 (CVCL_0031) and MDA-MB-231(CVCL_0062) human breast adenocarcinoma lines (European Cell and Culture Collection, Sailsbury, UK) were maintained in Dulbecco’s Modified Eagle’s Medium (DMEM) (ThermoFisher Scientific, Cramlington, UK) supplemented with 10% foetal bovine serum (FBS) (ThermoFisher Scientific) and 2 mM L-glutamine (ThermoFisher Scientific), whereas the MCF10-A (CVCL_0598) human breast epithelial line (American Type Culture Collection, Glasgow, UK) was maintained in a medium comprising Dulbecco’s Modified Eagle’s Medium/Nutrient Mixture F-12 (DMEM:F12) (ThermoFisher Scientific), 10% FBS, 0.5 µgmL^−1^ hydrocortisone (Sigma-Aldrich, Haverhill, UK), 10 µgmL^−1^ human insulin (Sigma-Aldrich), 20 ngmL^−1^ human epidermal growth factor (EGF) (Peprotech, London, UK) and 100 nM isoprenaline (Tocris, Avonmouth, UK).

All cell lines were incubated at 37 °C, 5% CO_2_ in a humidified environment.

### 2.2. Maintenance of Fibroblast Populations

Primary mammary fibroblasts (MFs) (Breast Cancer Now, Registered Charity No: 1160558, London, UK) were derived from healthy tissue obtained from a patient during a normal mammoplasty and maintained in DMEM:F12 supplemented with 10% FBS. Neonatal human dermal fibroblasts (HDFn, C0045C) (ThermoFisher Scientific) derived from male foreskin were cultured in DMEM supplemented with 10% FBS and 2 mM L-glutamine. Both populations were maintained up to 80% confluence at 37 °C, 5% CO_2_ in a humidified environment.

### 2.3. Cell Line Authentication

All cell lines described within this study were obtained at the start of the study in 2021 and were authenticated and screened from their sources. They have undergone routine mycoplasma testing and remained free from infection. These cell lines represent authenticated, commercially available, standard cell lines that have not previously been misidentified or contaminated.

### 2.4. Two-Dimensional Migration Assay

Breast epithelial cell lines (MCF-7, MCF10A and MDA-MB-231) were cultured to confluence in 12-well adherent culture plates (Greiner Bio-One, Stonehouse, UK) and serum-starved for 24 h prior to the assay being performed. Cultures were then treated with 5 µgmL^−1^ mitomycin C (Tocris, Bristol, UK) 2 h prior to commencement of the assay. Cells were washed in phosphate-buffered saline (PBS) to remove remaining mitomycin C, and a vertical scratch was made in each well using a 10 µL pipette tip. Phase contrast images of cultures were obtained over a 48 h period using the Zeiss Cell Observer. Three distinct areas along the scratch were imaged per well every 15 min over the 48 h period.

### 2.5. Three-Dimensional Alvetex^®^ Strata Migration Assay

The smaller pore variant, Alvetex^®^ Strata (Reprocell Europe, Glasgow, UK), was rendered hydrophilic through a wash in 70% ethanol, followed by PBS (Sigma-Aldrich). Breast epithelial cells were then seeded 7.5 × 105 cells per scaffold in a volume of 100 µL medium and allowed to adhere for 2 h at 37 °C, 5% CO_2_ in a humidified environment. Scaffolds were cultured within a standard 12-well culture plate in 4 mL of the relevant culture medium for a total of 14 days prior to analysis.

### 2.6. Stromal Compartment Construction

The larger pore variant, Alvetex^®^ Scaffold (Reprocell Europe), in a 24-well format, was used to construct stromal and full-thickness engineered tissues. The scaffolds were first rendered hydrophilic through ethanol washing prior to use. Either HDFn or MF cells were seeded at a density of 1.7 × 105 cells per scaffold in a volume of 50 µL and allowed to adhere for 2 h at 37 °C, 5% CO_2_ in a humidified environment. Following this, 3 mL of the relevant culture medium supplemented with 100 µgmL^−1^ ascorbic acid (Sigma-Aldrich) and 5 ngmL^−1^ TGFβ1 (Peprotech) was added to each scaffold and maintained for 14 days with twice-weekly media changes to promote neosynthesis of ECM.

### 2.7. Full-Thickness Epithelial–Stromal Breast Cancer Invasion Model

Stromal compartments were engineered as above and matured for 14 days. Each breast epithelial cell population (MCF-7, MCF-10A and MDA-MB-231) was seeded onto stromal compartments at a density of 1.5 × 10^6^ cells per scaffold and allowed to adhere for 2 h at 37 °C, 5% CO_2_ in a humidified environment.

### 2.8. Caffeic Acid Phenethyl Ester (CAPE) Inhibition

Both Alvetex^®^ Strata and full-thickness epithelial–stromal invasion models were supplemented with either 10 µM CAPE (Selleckchem, TX, USA) or DMSO (Sigma-Aldrich) as a vehicle-matched control for 14 days prior to harvest.

### 2.9. Paraffin Wax Embedding

3D tissue constructs were harvested and fixed in 4% paraformaldehyde (PFA) (Sigma-Aldrich) overnight at 4 °C. Once fixed, scaffolds were washed twice in PBS and dehydrated through a series of ethanols (30–100%) and embedded in paraffin wax as previously described [45,48,49]. Paraffin wax blocks were then sectioned transversely at 5 μm using a microtome (Leica RM2125RT; Leica, Wetzlar, Germany) and mounted onto charged microscope slides (ThermoFisher Scientific).

### 2.10. Haematoxylin and Eosin (H&E) Staining

Samples were deparaffinised in histoclear and stained as previously described [45,48,49]. H&E-stained samples were imaged using a Leica ICC50 high-definition camera and Brightfield microscope and images were captured using Leica EZ software.

### 2.11. Immunofluorescence

Both 2D and 3D cultures were immunostained as previously described [45,48,49]. Primary antibody (Table S1) diluted in blocking buffer was then added to samples for one hour at room temperature and incubated overnight at 4 °C in a solution of the relevant secondary antibody (AlexaFluor^®^ anti-mouse 488 nm (ThermoFisher Scientific), AlexaFluor^®^ anti-rabbit 594 nm (ThermoFisher Scientific)) diluted in blocking buffer with the nuclear dye Hoescht-33342 (ThermoFisher Scientific). Immunofluorescently labelled samples were imaged using a Zeiss 880 confocal microscope with Zen software (Carl Zeiss, Cambridge, UK).

### 2.12. Image Analysis

Image J software (NIH, Bethesda, MD, USA) was used to measure cellular migration in both 2D and 3D culture systems. For 2D samples, a grid overlay was applied to phase contrast images of isolated time points (T = 0 h, T = 48 h). The edge of the scratch wound was established using the free-hand line tool and measurements were taken horizontally along the partitions. Data were expressed as a percentage of T = 0.

Cellular invasion in 3D culture samples was measured by the same method, but vertical measurements of penetration were made along the scaffold and were expressed as a percentage of the 200 µm thick scaffold.

### 2.13. Statistical Analysis

GraphPad Prism v10 (GraphPad Software, San Diego, CA, USA, www.graphpad.com) was used to determine statistical significance, with details on the choice of statistical test detailed in individual figure legends. Differences were considered significant at *p* < 0.05.

## 3. Results

### 3.1. Characterisation of Breast Cancer Lineages

Traditional approaches to modelling migratory potential in vitro centre around the scratch wound healing assay. Cells are cultured in a monolayer in which a “scratch” is made along the centre, and either the time taken for the wound to close or the distance migrated by cells is measured. This offers an inexpensive, well-characterised, high-throughput approach, as described in the literature [50,51,52]. In this study, we applied this approach to three extensively studied breast cell lines whose characteristics are representative of different cancer subtypes. The MCF-10A line represents a non-tumorigenic immortalised cell line, isolated from fibrocystic breast tissue, whereas the MCF-7 line is tumorigenic and widely considered a moderately invasive representation of hormone-dependent breast cancer (ER+), and the MDA-MB-231 population displays a triple-negative, aggressive and invasive phenotype [53,54,55].

**Figure 1:**
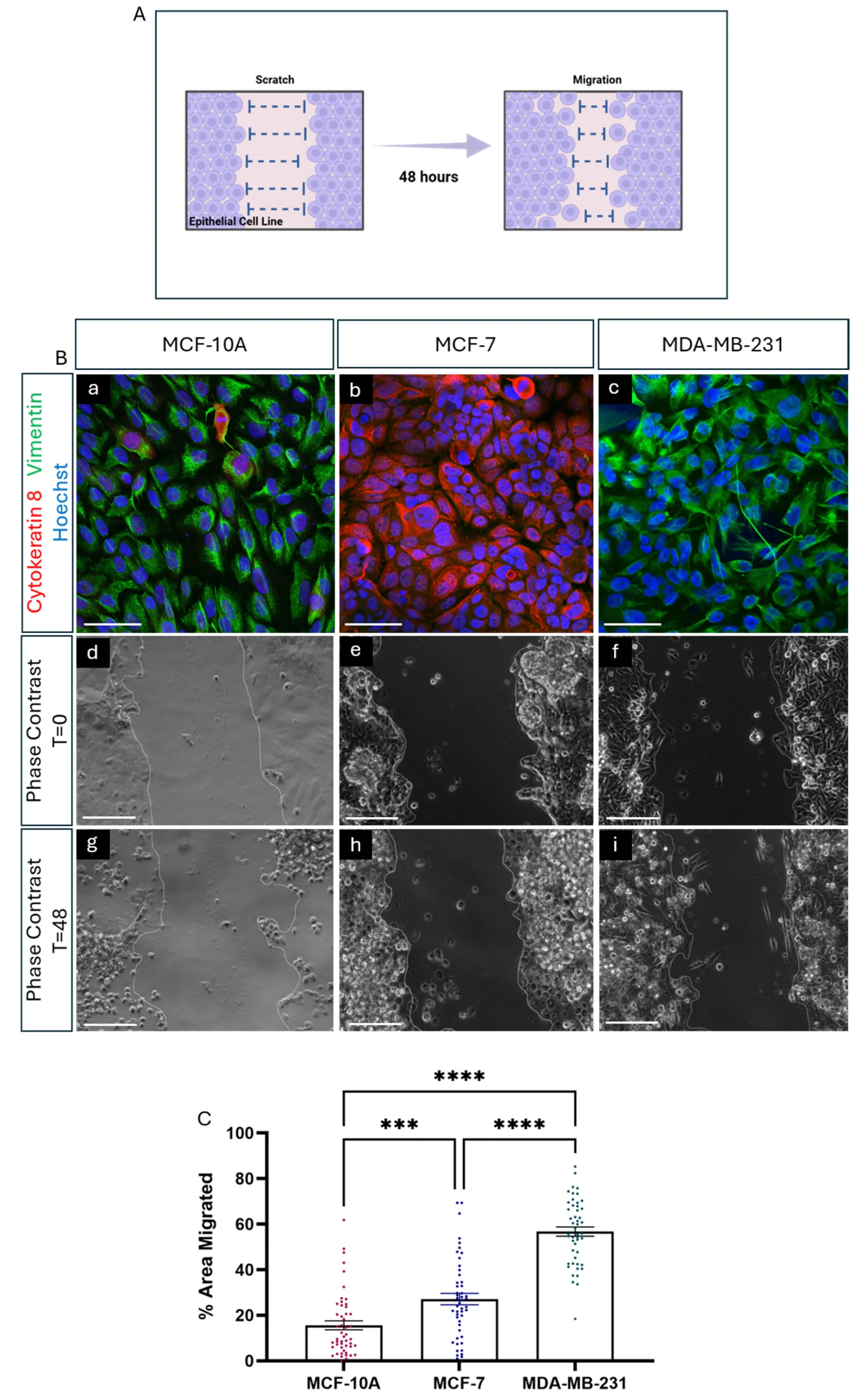
Conventional two-dimensional approaches identify differences in epithelial breast cancer cell migratory potential. Schematic representation of the scratch assay methodology, in which a 2D monolayer of epithelial cells is scratched and migration assessed over a 48 h period (dotted line) (**A**). Immunofluorescent comparison of cytokeratin 8 (red) and vimentin (green) expression between the MCF-10A, MCF-7, and MDA-MB-231 breast epithelial cell lines with differing migratory capacities. Nuclei are highlighted in blue (**B**(**a**–**c**)). Phase contrast micrographs representing scratch assays conducted for each epithelial cell line at 0 h (**B**(**d**–**f**)) and 48 h post-scratch (**B**(**g**–**i**)). Borders of the scratch are marked with a solid white line. Quantification of scratch closure as percentage area migrated by epithelial cells following 48 h (**C**) (data represent mean ± SEM, *n* = 5) reveals significant differences between cell lines. One-way ANOVA, *** = *p* < 0.001, **** = *p* < 0.0001. Scale bars: (**B**(**a**–**c**)): 50 µm, (**B**(**d**–**i**): 25 µm.

Although the migratory potential of each cell line has been described extensively throughout the literature, it is known to be highly dependent upon culture conditions. A thorough assessment utilising the traditional scratch assay approach was conducted to provide an important benchmark with which increasingly complex culture systems could be evaluated. This provides a baseline capable of demonstrating any differences in cellular behaviour when cultured in a 3D environment or in the presence of supporting cell types. Each epithelial cell line was cultured as a monolayer and wounded, and the resultant culture was imaged in real time over a 48 h window, and migration was calculated (Figure 1A).

Immunoreactivity of two biomarkers consistent with epithelial (cytokeratin 8 (CK8)) and mesenchymal (vimentin) cells revealed two distinct subpopulations within the MCF-10A monolayer (Figure 1B(a)), with only a small population of cells positive for CK8 and the majority of cells vimentin-positive. As it is a model of non-invasive breast epithelia, high levels of CK8 expression are expected. However, it is well documented that, particularly when under physical stress, vimentin expression is inducible in this population. Therefore, artificially high vimentin expression could be an artefact of the 2D culture environment. However, the MCF-7 population, representative of tumorigenic but non-invasive breast cancer, demonstrated significant positive immunoreactivity to CK8 and a complete lack of vimentin staining (Figure 1B(b)). This finding is consistent with its invasive potential and suggests cells have not undergone epithelial–mesenchymal transition (EMT) 46 [56,57]. In contrast, MDA-MB-231 cells expressed high levels of vimentin, but lacked any discernible CK8 positivity, suggesting the cells have fully undergone EMT, which is consistent with their highly invasive nature (Figure 1B(c)).

Scratch assays were performed on 2D confluent monolayers of each cell line that had been incubated with mitotic inhibitors to eliminate the impact of cell proliferation on scratch closure (Figure 1B(d–f)). After 48 h in culture, there was little noticeable difference in scratch width for the MCF-10A population (Figure 1B(g)), compared with a small observable reduction in scratch width for the MCF-7 cell line (Figure 1B(h)) and an obvious reduction in scratch width for the MDA-MB-231 line (Figure 1B(i)). This is supported by quantification, which details a small percentage area migrated by MCF-10A, a significantly greater area migrated by MCF-7, and an increased area migrated by MDA-MB-231 that was significantly greater than that of either of the other cell lines tested (Figure 1C). These data are consistent with the known characteristics of each cell line, but 2D scratch assays are limited due to their restricted geometry and limited cell–microenvironmental interactions, which, via mechanotransduction, can be responsible for changes in cell function, including migration [58,59].

### 3.2. Development of a Simple 3D Cancer Migration Assay In Vitro

**Figure 2:**
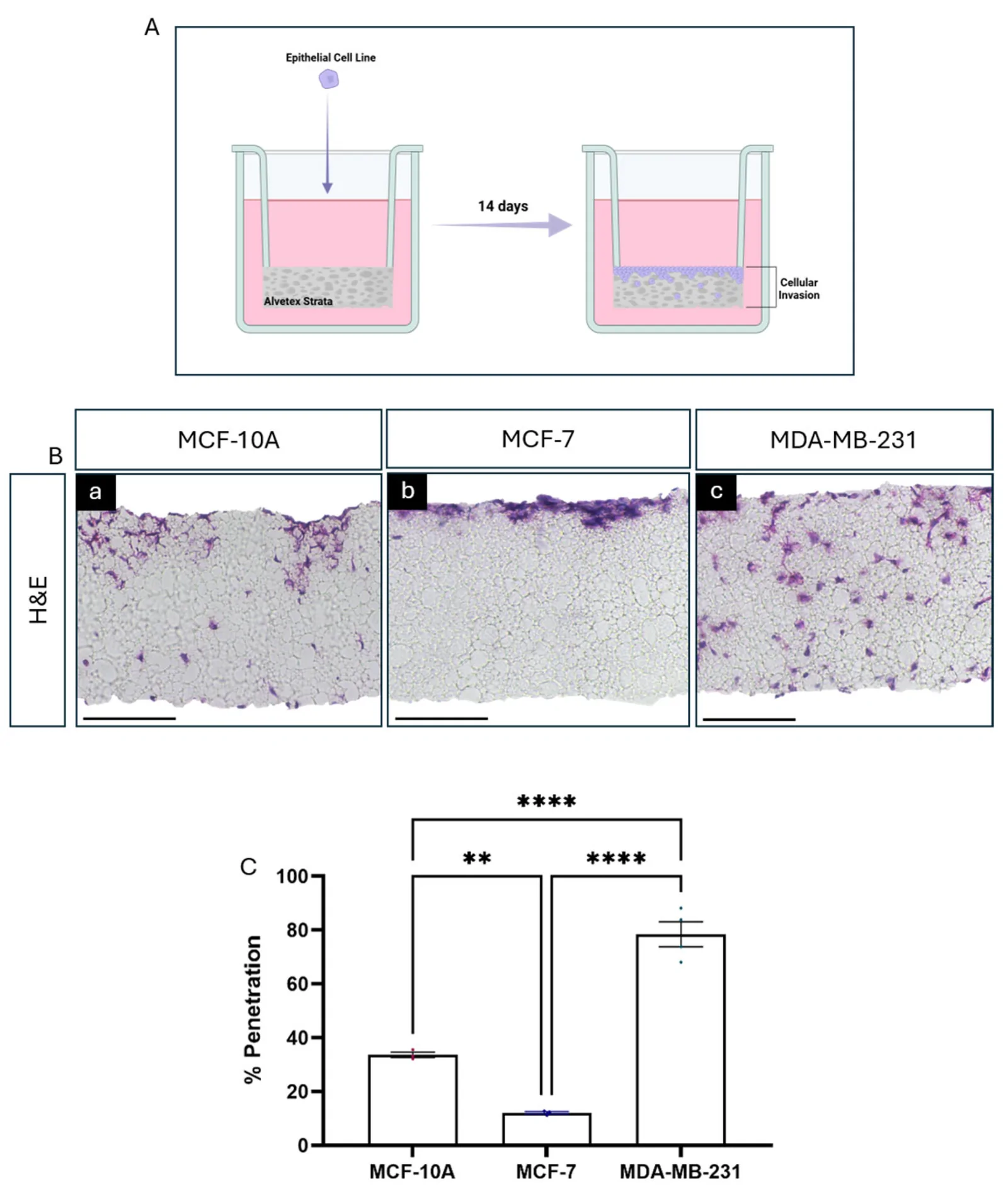
Introduction of a three-dimensional culture geometry provides a more physiological approach to assess breast cancer cell migration in vitro. Schematic representation of the culture methodology, in which epithelial cells were seeded onto Alvetex^®^ Strata (average pore size: 15 µm) and cultured for 14 days prior to assessment of cellular invasion within the 3D scaffold (**A**). Histological analysis reveals the extent of cellular migration throughout the 3D material for each breast epithelial cell line, with moderate infiltration observed for MCF-10A, very little invasion noted for MCF-7, and MDA-MB-231 demonstrating population of the entire scaffold (**B**). Quantification of percentage penetration of the cell lines throughout the scaffold reveals stark differences between each of the three lines tested (data represent mean ± SEM, *n* = 3) (**C**). One-way ANOVA, ** = *p* < 0.01, **** = *p* < 0.0001. Scale bars: 100 µm.

To improve the assay’s physiological relevance and better recapitulate the 3D geometry that cancer cells experience in vivo, a 3D culture environment was introduced to each of the three cell lines to elevate the complexity of the in vitro system. Each epithelial cell line was seeded onto Alvetex^®^ Strata, the smaller pore size variant (~15 µm), and cultured for 14 days prior to quantification of cell penetration (Figure 2A). Histological analysis reveals moderate penetration of MCF-10A (Figure 2B(a)) and little to no invasion of the 3D material by MCF-7 cells (Figure 2B(b)). This differs from the MDA-MB-231 line, which fully populated the scaffold, with cells visible throughout the 3D material, including the basal surface (Figure 2B(c)). Quantification supports these observations, with moderate penetration observed with the MCF-10A line, significantly less penetration observed with MCF-7 and significantly more penetration by MDA-MB-231 compared with either of the other cell lines (Figure 2C).

Although a 3D geometry provides cells with mechanical cues, the cancer microenvironment also consists of a multitude of other complex interacting factors: for example, biochemical interactions with the ECM, physical contact and paracrine communication between cell types. These are concepts that we have previously explored in bioengineered tissues [60,61,62,63,64,65,66]. To better recapitulate the complex communication between cell types in the native breast tissue environment, we engineered a stromal compartment beneath the epithelium, rich in endogenous ECM proteins, to further enhance the physiological relevance of this system as a potential predictive tool.

### 3.3. Impact of Stromal–Epithelial Interactions When Modelling Cancer Invasion In Vitro

**Figure 3:**
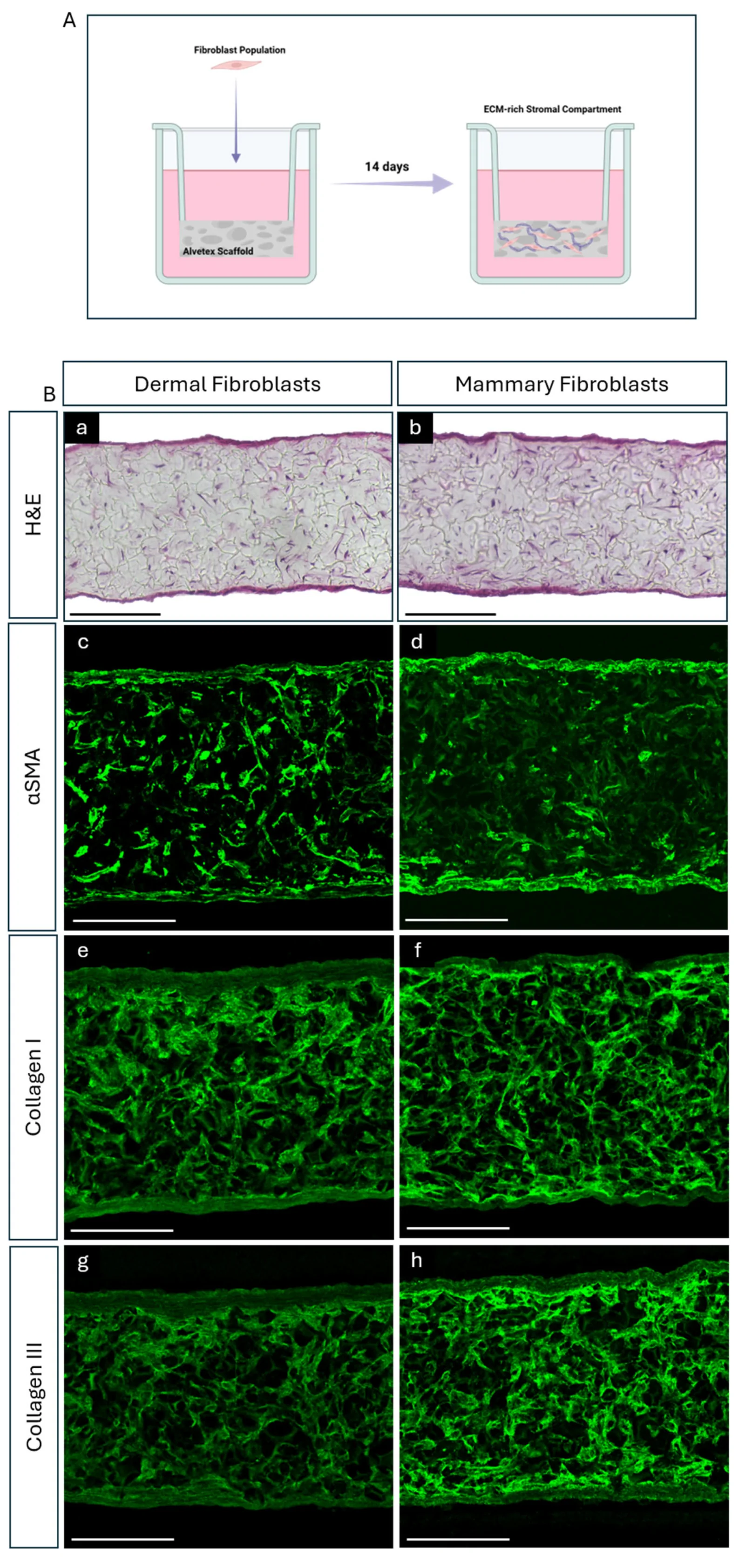
Development of a mammary-tissue-specific stromal compartment in vitro. Schematic representation of the construction of stromal compartments derived from primary dermal or mammary fibroblasts. Fibroblasts were seeded within Alvetex^®^ Scaffold (average pore size: 40 µm) and cultured for 14 days to promote cellular population of the scaffold and neosynthesis of endogenous extracellular matrix (ECM) (**A**). Histological analysis reveals population of the 3D material with each fibroblast population (**B**(**a**,**b**)). Immunofluorescence detection of αSMA (**B**(**c**,**d**), green), a biomarker consistent with the myofibroblast phenotype, and key components of the ECM, collagens I (**B**(**e**,**f**), green) and III (**B**(**g**,**h**), green), demonstrates key biochemical signatures specific to stromal compartments engineered from each fibroblast population. Scale bars: 100 µm.

Previously, we demonstrated the impact of stromal–epithelial interactions on tissue structure and function in bioengineered intestine and skin models, with an emphasis on the role of tissue-specific fibroblast populations [42,45]. For this reason, we initially characterised the growth of dermal and mammary fibroblasts in 3D and their resultant biomarker expression. To generate a functional stromal compartment, Alvetex^®^ Scaffold was used, as the larger pore size (~40 µm) is amenable to fibroblast populations. Each fibroblast subtype was seeded into Alvetex^®^ Scaffold and cultured for 14 days, during which time the cells populated the scaffold and neosynthesised endogenous ECM (Figure 3A).

Histological analysis revealed that both dermal (Figure 3B(a)) and mammary fibroblasts (Figure 3B(b)) successfully populated the 3D material lining both the apical and basal surfaces. Immunofluorescent analysis of αSMA, a key characteristic of CAFs [18,19,21,23], revealed significant expression in both populations (Figure 3B(c,d)), likely due to TGFβ supplementation; however, αSMA expression alone is insufficient to label the populations CAFs. In both cases, there was evidence of a dense neosynthesised ECM secreted endogenously by the fibroblasts. The expression of both collagens I (Figure 3B(e,f)) and III (Figure 3B(g,h)) appeared increased in mammary fibroblast-derived stromal compartments through qualitative observation, but in the future proteomic analysis would help to quantify these differences. The architecture and composition of ECM are particularly important, as interactions with the ECM and remodelling events impact cancer progression and prognosis [23,67,68].

**Figure 4:**
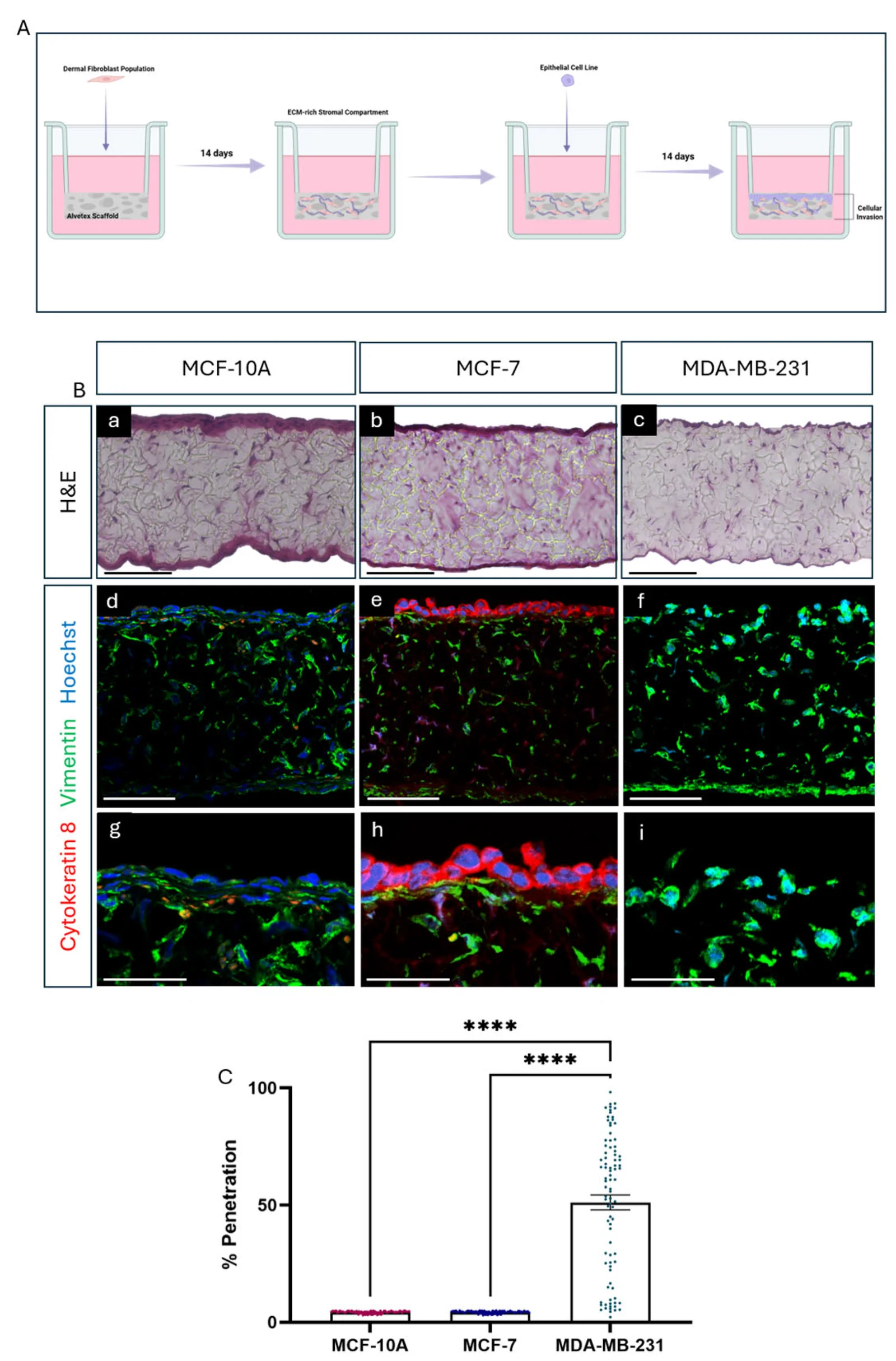
Co-culture of breast cancer epithelial lines with dermal fibroblasts results in a bi-layered construct capable of modelling epithelial–stromal interactions. Dermal fibroblasts were seeded within Alvetex^®^ Scaffold for 14 days to allow for population of the scaffold and production of endogenous extracellular matrix (**A**). Breast epithelial cell lines were then seeded onto mature submucosal compartments and cultured for a further 14 days to prior to assessment of invasion. Histological assessment demonstrates population of the 3D scaffold with dermal fibroblasts (**B**(**a**–**c**)), and MCF-10A and MCF-7 epithelial cells can be identified to have formed a distinct epithelial compartment apical to the submucosal compartment, whereas in the case of MDA-MB-231 cells there is no obvious compartmentalisation between the cell types. Immunofluorescent staining for cytokeratin 8 (red) and vimentin (green) (**B**(**d**–**f**)) provides a more detailed depiction of the interaction between stromal and epithelial cell types, including high-magnification imaging of epithelial compartments (**B**(**g**–**i**)). Nuclei are stained by Hoechst (blue). Quantification of percentage penetration (**C**) of the scaffold by each epithelial cell line demonstrates the highly invasive nature of MDA-MB-231 (data represent mean ± SEM, *n*= 9). One-way ANOVA, **** = *p* < 0.0001. Scale bars: (**B**(**a**–**f**)):100 µm, (**B**(**g**–**i**)): 50 µm.

Initial optimisation attempts focussed on the interactions between stromal compartments engineered from dermal fibroblasts and each of the three characterised breast epithelial lines. Stromal compartments were generated by seeding dermal fibroblasts into Alvetex^®^ Scaffold and culturing them for 14 days to promote ECM deposition. Each breast epithelial cell line (MCF-10A, MCF-7 and MDA-MB-231) was then seeded onto the stromal construct and cultured for a further 14 days prior to assessment of invasion (Figure 4A).

Histological analysis revealed a notable lack of invasion of MCF-10A cells. Epithelial cells appeared in a defined compartment apical to the stromal compartment (Figure 4B(a)). This observation was confirmed through immunodetection of CK8 and vimentin, which demonstrated the presence of only a small number of CK8-positive cells that had migrated into the vimentin-rich stromal compartment (Figure 4B(d,g)). This indicates confinement of MCF-10A to the epithelial compartment and more clearly reflects the organisation of healthy mammary tissue.

Similarly, MCF-7 cells were clearly compartmentalised to the apical surface (Figure 4B(b)), as confirmed by immunodetection of CK8, indicating little invasion into the vimentin-positive stromal compartment (Figure 4B(e)). Rather than appearing as a monolayer atop the stromal compartment, MCF-7 cells appear multilayered in places (Figure 4B(e,h)). This is consistent with non-invasive, confined lesions; however, other pathological hallmarks such as absence of myoepithelial cells and basement membrane components would further support this observation. These findings suggest the presence of the stromal compartment has impacted the morphology of the epithelial cells, better recapitulating the in vivo state.

In contrast, when the highly invasive MDA-MB-231 line was cultured on a stromal compartment engineered from dermal fibroblasts, there was no evidence of a defined epithelial layer (Figure 4B(c)). As MDA-MB-231 cells undergone EMT and express vimentin, immunostaining of the construct revealed the presence of epithelial cells throughout the stromal compartment, identifiable by their distinct morphology (Figure 4B(f,i)). This demonstrates the invasive nature of MDA-MB-231 cells, which had migrated into the scaffold despite dense population by both fibroblasts and ECM. Quantification confirms these observations, with only a small percentage of cells having penetrated the 3D material in the cases of both MCF-10A and MCF-7 cells, whereas penetration was significantly higher for the MDA-MB-231 line (Figure 4C).

**Figure 5:**
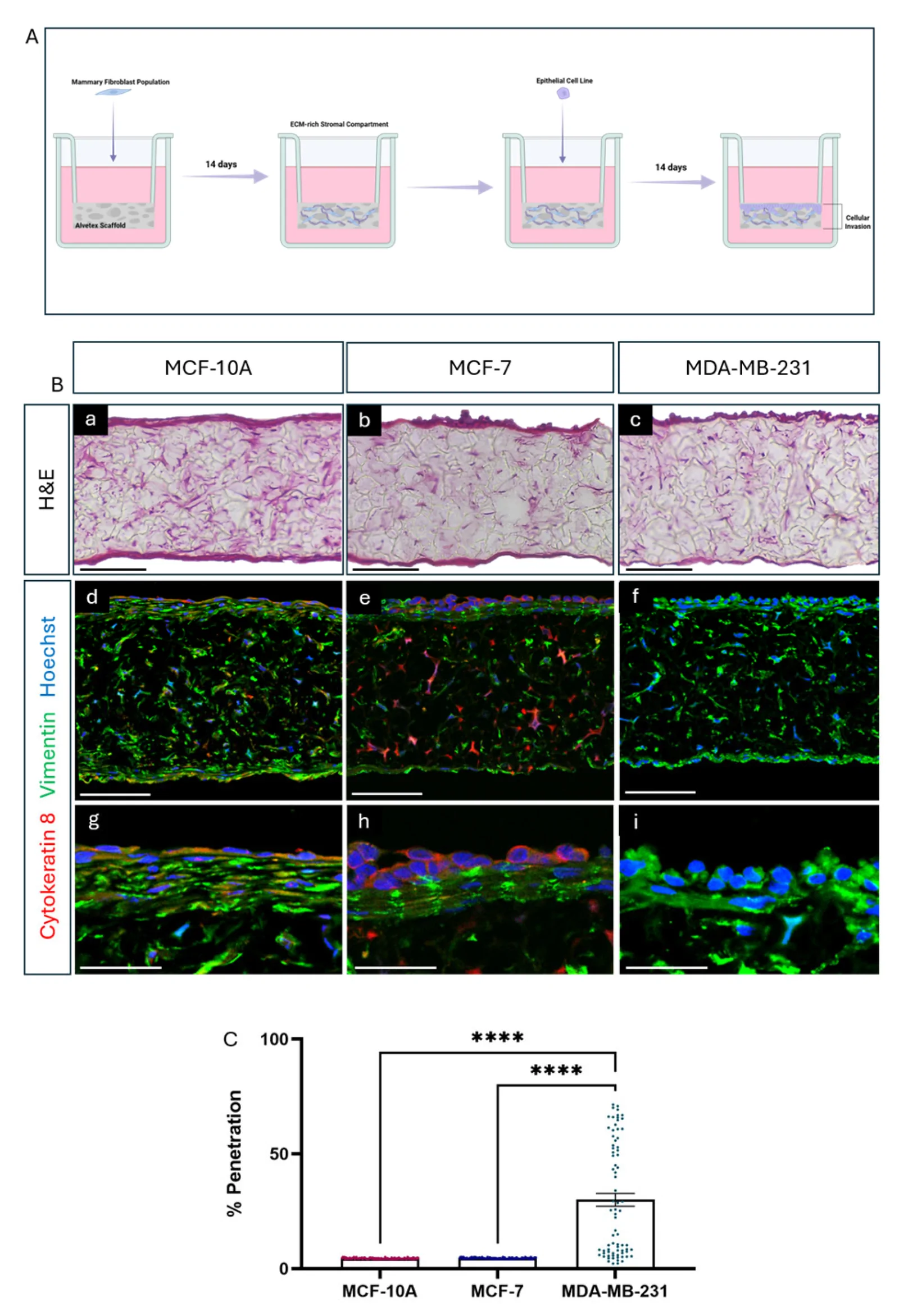
Stromal compartments engineered from tissue-specific fibroblasts impact epithelial compartment structure in vitro. Mammary fibroblasts were seeded within Alvetex^®^ Scaffold for 14 days, during which time cells populated the scaffold and neosynthesised tissue-specific extracellular matrix (**A**). Breast epithelial cells were then seeded onto mature stromal compartments and cultured for a further 14 days to assess their invasive potential. Histological staining (**B**(**a**–**c**)) in all cases depicts a stromal compartment populated by mammary fibroblasts with a distinct epithelial compartment comprising either MCF-10A, MCF-7 or MDA-MB-231 breast epithelial cells. Immunofluorescent staining (**B**(**d**–**f**)) of biomarkers consistent with epithelial or fibroblast lineages reveals cytokeratin 8 (red) positivity and vimentin (green) expression, with high-magnification focus on the epithelial compartment (**B**(**g**–**i**)). Nuclei are stained in blue by Hoechst. Percentage penetration into the stromal compartment by each epithelial cell line reveals differences in their invasive potential (data represent mean ± SEM, *n*= 9) (**C**). One-way ANOVA, **** = *p* < 0.0001. Scale bars: (**B**(**a**–**f**)):100 µm, (**B**(**g**–**i**)): 50 µm.

In the past we have demonstrated functional differences in bioengineered constructs assembled from tissue-specific fibroblast subtypes [42,45]. For this reason, we then incorporated mammary fibroblasts to produce a more complex and tissue-specific system. As with their dermal counterpart, mammary fibroblasts were seeded into the 3D scaffold and cultured for 14 days, during which time they secreted endogenous ECM. Epithelial cells were then seeded onto mature stromal compartments and cultured for a further 14 days prior to invasion analysis (Figure 5A).

Interestingly, MCF-10A cells appeared as a histologically distinct compartment, similar to culture on a dermal fibroblast-based stromal compartment (Figure 5B(a)). However, immunofluorescence analysis revealed increased CK8 positivity, with a distinct change in epithelial cell morphology: cells appeared more elongated but were confined to the apical surface of the stromal compartment (Figure 5B(d,g)).

At the histological level, MCF-7 cells visibly populated the surface of the construct, but unlike when cultured on a dermal fibroblast-rich stromal compartment, appeared up to 4-5 cell layers thick in places (Figure 5B(b)). This finding is corroborated by the detection of CK8-positive cells only in the epithelial compartment, denoting a lack of invasion into the vimentin-rich stroma, which appear cuboidal and multilayered (Figure 5B(e,h)). However, there are notable differences when compared with the dermal fibroblast-based construct, indicative of a differential response likely due to communication between the cell types.

When cultured on a stromal foundation of mammary fibroblasts, an epithelial compartment comprising MDA-MB-231 cells was identifiable (Figure 5B(c)), unlike the complete invasion observed when cultured on a dermal-fibroblast-based stromal compartment. Vimentin-positive MDA-MB-231 cuboidal cells were stacked in a multilayered epithelium above the stromal compartment (Figure 5B(f,i)). However, some invasion of MDA-MB-231 cells was detected in the stromal layer beneath the epithelium (Figure 5C). These findings demonstrate the invasive nature of these cells, a phenotype suggestive of EMT.

### 3.4. Inhibition of Breast Cancer Cell Invasion by Caffeic Acid Phenethyl Ester (CAPE)

Caffeic acid phenethyl ester (CAPE) is a small molecule that has been extensively reported in the literature to reduce cancer cell invasion and induce apoptosis specifically in the MCF-7 and MDA-MB-231 cell lines [69,70]. It has been demonstrated to inhibit EMT in the breast cancer lineages described in this study [71], modulate Bcl-2 and caspase activity to trigger the apoptotic cascade and possess antioxidant and anti-tumorigenic properties though inhibition of NFκB-mediated signalling [70,72]. For this reason, we applied CAPE to both the simple Strata and more complex full-thickness invasion assays described in this study to demonstrate their potential as screening platforms. This proof-of-concept trial utilises CAPE as a reference compound whose mechanism of action is well understood, demonstrating the translational nature of the model systems described throughout this study.

**Figure 6:**
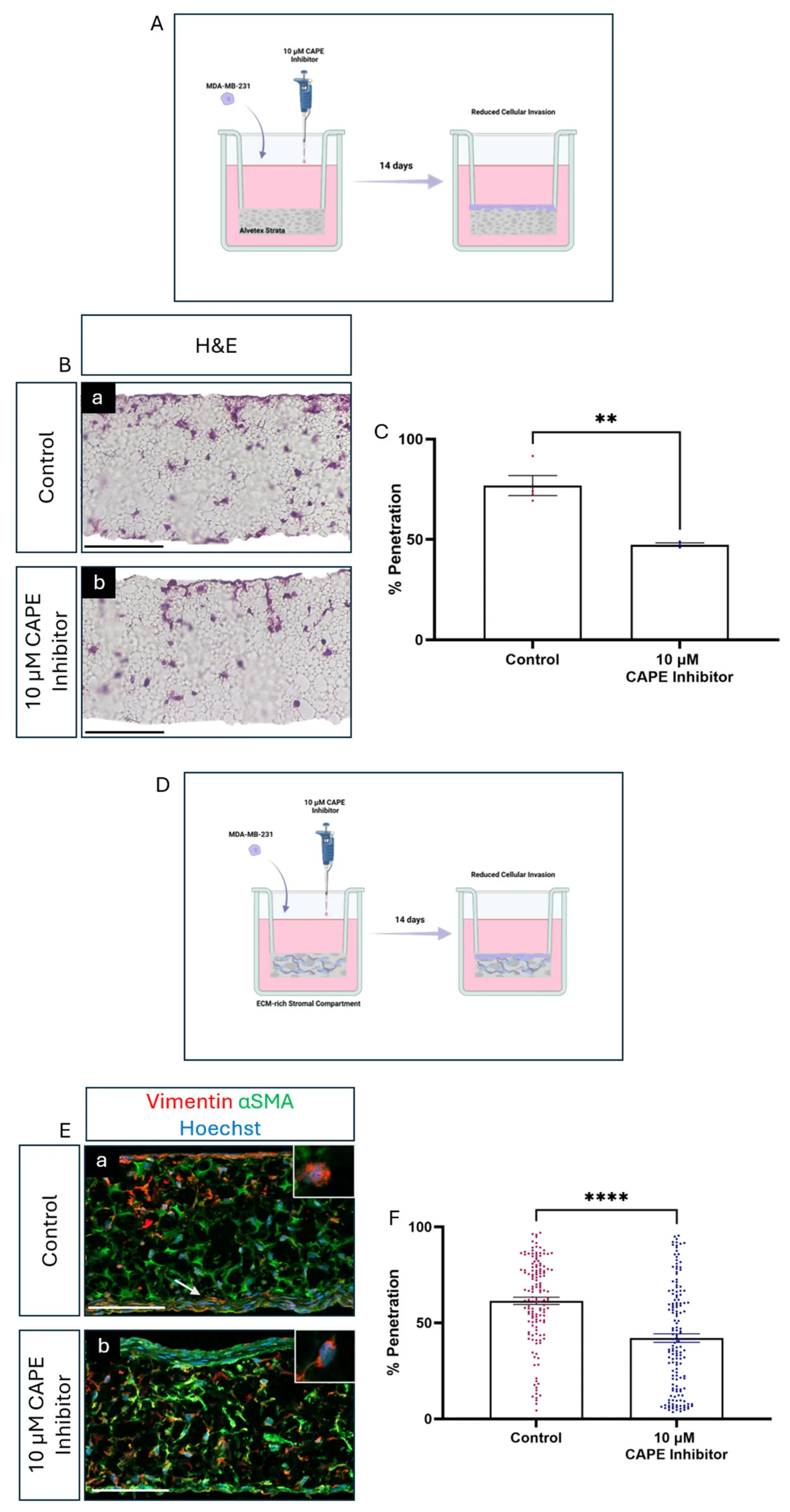
Caffeic acid phenethyl ester (CAPE) inhibits breast cancer cell invasion in a complex bioengineered invasion platform. Schematic representation of the three-dimensional cancer cell invasion assay (**A**), in which the invasive MDA-MB-231 cells were seeded onto Alvetex^®^ Strata in medium supplemented with either DMSO (vehicle control) or 10 µM CAPE and cultured for 14 days. Representative histological images demonstrate both cellular penetration into the scaffold in the absence (**B**(**a**)) of CAPE inhibition and reduced penetration in the presence of CAPE inhibition (**B**(**b**)). Quantification of percentage penetration (**C**) into the scaffold reveals a significant reduction when treated with 10 µM CAPE for 14 days (data represent mean ± SEM, *n* = 3). Schematic representation of the more complex co-culture methodology (**D**) involved the pre-seeding of mammary fibroblasts into Alvetex^®^ Scaffold allowing for the secretion of endogenous extracellular matrix to provide a stromal foundation prior to invasion assessment. The invasive MDA-MB-231 line was then seeded onto the stromal foundation supplemented with either DMSO (vehicle control) or 10 µM CAPE and cultured for 14 days. Immunofluorescence analysis (**E**) allows for distinction between the vimentin (red)-positive epithelial cells (arrow) and αSMA (green)-positive fibroblast population, with nuclei highlighted in blue by Hoechst. Quantification of percentage penetration into the stromal compartment is significantly reduced in constructs treated with 10 µM CAPE (data represent mean ± SEM, *n* = 9) (**F**). Student’s *t*-test, ** = *p* < 0.01, **** = *p* < 0.0001. Scale bars: 100 µm.

The highly invasive MDA-MB-231 cell line was cultured for 14 days in the presence of 10 µM CAPE (Figure 6A). Histological analysis revealed significant invasion of cells into the 3D material, with cells visible throughout the scaffold (Figure 6B(a)). However, when cultured in the presence of CAPE, penetration into the 3D material was visibly reduced, with fewer cells reaching the basal surface of the scaffold having penetrated the entire depth (Figure 6B(b)). This was confirmed through quantification of penetration of cells, which was significantly reduced when treated with 10 µM CAPE (Figure 6C).

The impact of CAPE-mediated inhibition of cell invasion was probed in the more complex full-thickness invasion model, which included a mammary fibroblast-based stromal compartment. Firstly, the stromal compartment was matured for 14 days, followed by seeding of MDA-MB-231 cells in the presence of 10 µM CAPE for 14 days (Figure 6D). Immunofluorescence detection was used to distinguish between epithelial and fibroblast cell types. As MDA-MB-231 cells express vimentin as opposed to CK8, this alone was not sufficient to identify them amongst a population of fibroblasts; therefore, co-localisation of αSMA and vimentin served to differentiate cell types. MDA-MB-231 cells are well known to express vimentin but not αSMA, and early characterisation attempts demonstrated this in our culture conditions; therefore, this expression profile, combined with cell morphology observations, was sufficient to distinguish between epithelial and stromal populations for this purpose. However, in the future, more confirmatory analyses, such as lineage-tracing dyes, should be applied to provide an additional discriminatory feature. Vimentin-positive, αSMA-negative cells (insert, indicative of MDA-MB-231) were observed to have penetrated the entirety of the stromal compartment and were visible on the basal surface of the construct (arrow, Figure 6E(a)) in control conditions. The presence of 10 µM CAPE in the culture medium reduced the number of such cells visible on the basal surface (Figure 6E(b)). This was confirmed through quantification, which revealed a significant reduction in cell penetration into the stromal compartment in the presence of CAPE inhibition of invasion (Figure 6F).

## 4. Discussion

Interactions between cells and their surrounding microenvironment play an integral role in tissue homeostasis and functionality [73]. Neoplastic cellular change coupled with microenvironmental interactions are thought to be major contributing factors to cancer cell migration, metastasis and prognosis [17,74,75]. However, accurately replicating the cancer microenvironment in vitro remains challenging for bioengineers. This is problematic, as the predictive capacity of current model systems is often limited due to limitations in the technological approach. For this reason, there is a need for improved cellular approaches of increasing complexity, capable of recapitulating microenvironmental factors that contribute to metastasis and cancer progression.

Cancer cell migration is a crucial factor in disease progression, as it enables cells to leave their primary site, invade the surrounding tissues and traverse the body. This process of metastasis is the leading cause of cancer-related deaths (~90%) and results in difficulties delivering targeted treatments [76,77]. Alongside increased proliferative capacity, enhanced migratory potential is considered a key feature of malignant transformation; as cells undergo EMT they lose epithelial traits and become more mesenchymal, whilst their motility and invasiveness increase [56,78]. Migratory ability is governed not only by intrinsic changes, but also by how amenable the surroundings are to migration. Microenvironmental features permissive to cellular migration include ECM remodelling governed by cancer-associated fibroblasts (CAFs) which secrete matrix metalloproteinases (MMPs) [19,23]. These changes to the cancer microenvironment support cancer cell migration through changes to the architecture, mechanics, oxygen content, and paracrine signalling, which fuel angiogenesis, invasion, immune evasion and metastatic potential. This highlights the need to understand cancer cell biology in the context of their microenvironment through interactions with their neighbouring stromal cells, ECM and cell signalling.

Current in vitro approaches lack this level of complexity, with the most widely described approach to assessing invasive potential still being the scratch assay originally pioneered in the 1960s [50]. 2D cell culture techniques force cells into an artificial environment, resulting in flattened cell structure and abnormal nuclear morphology that impacts gene and protein expression whilst ultimately changing cell function [58,79,80,81]. These altered characteristics observed in 2D cell cultures are particularly problematic when inferring predictive value [47].

This study has presented a more complex, 3D culture system that models several aspects of the breast cancer microenvironment whilst overcoming some of the limitations associated with other 3D in vitro techniques, such as use of animal-derived products, lack of reproducibility, monoculture, etc. [66]. Within this system we have utilised commonly described breast epithelial cell lines with various invasion capacities: MCF-10A, an immortalised non-tumorigenic cell line, MCF-7 HR+ cells with limited invasion potential, and finally the more invasive MDA-MB-231 triple-negative cells [54]. Through adoption of a 3D culture geometry, the cells were afforded a larger surface area to migrate, more akin to the 3D tissue environment in vivo, therefore improving physiological relevance. This is particularly important as cancer invasion into the mesenchymal compartment is an important factor when staging and assessing disease progression [78,82]. Through providing the cell lines with a physical compartment through which to migrate, their migratory potential can be probed in a manner more reflective of the in vivo state. In this simplistic 3D system, MCF-7 cells were observed to form a multilayered tissue atop the scaffold, whilst MDA-MB-231 cells were found to disperse throughout the scaffold, demonstrating stark differences in invasion [28,54,55].

Interactions between the stromal and epithelial compartments are an essential factor in cancer progression, due in part to the effects of CAFs on the neighbouring epithelial compartment [19]. A major obstacle in the development of tumour progression models is recapitulation of tissue-specific ECM in a density and proportion reflective of in vivo tissue. Many current approaches utilise animal-derived or undefined ECM components [34,35,53,83]; our approach centres on neosynthesis of endogenous human tissue-specific ECM in a cell-dependent manner. Compartmentalisation of a fibroblast/ECM-rich stromal foundation allowed for more accurate recapitulation of mammary tissue microanatomy.

Metastasis relies heavily on EMT and invasion of cancer cells from the epithelial compartment to the stromal compartment, which provides access to the circulatory and lymphatic systems, aiding their movement [25]. For this reason, the inclusion of a physiologically representative stromal compartment proves a valuable addition to the 3D invasion assay described in this study. The non-invasive behaviour of both the MCF-10A and MCF-7 lines demonstrates the development of a distinct epithelial compartment, contrasted by MDA-MB-231, which fully invaded the 3D material despite the presence of a dense network of ECM and fibroblasts. These findings emphasise the aggressively migratory phenotype of MDA-MB-231 cells. MCF-10A cells developed a typical elongated morphology in a defined epithelial compartment, like the MCF-7 cells, which, although more cuboidal in morphology, also produced a defined compartment; but unlike that of MCF-10A, theirs was multilayered [54]. The lack of invasion of MCF-7 and MCF-10A cells suggests the presence of a dense ECM network is sufficient to prevent invasion. However, as a model of invasive metastatic cancer, MDA-MB-231 cells did invade the stromal compartment across all platforms, which is unsurprising given that this cell line has been documented to highly express a broad range of MMPs, allowing them to degrade collagens, aiding their motility [18].

The presence of tissue-specific fibroblast subtypes impacts epithelial cell behaviour in this system, which is supported by previous findings [45]. Both dermal and mammary fibroblast populations were treated with TGFβ and expressed high levels of αSMA; given that TGFβ stimulation is a key signalling event in CAF development, this may enhance their disease-specific relevance, although further expression profiles would confirm this. When cultured with mammary fibroblasts, each cell type appears to exhibit more physiologically representative features. MCF-7 cells appeared more cuboidal and multilayered when cultured with mammary fibroblasts, and MDA-MB-231 cells were less invasive when co-cultured with mammary fibroblasts, perhaps in part due to the increased density of ECM deposition in comparison to the dermal fibroblast population. However, although they were obtained from different body locations, other key variables do differ between the fibroblast populations, such as age and sex. It is therefore impossible to determine if the differential phenotypes observed are a direct consequence of origin tissue or other factors without replicating the study with cells from a large variety of donors to account for biological variation. Unfortunately, that remains outside the scope of this study, particularly as primary cell availability was a limiting factor.

There are many factors that influence stromal–epithelial communication, including paracrine release of soluble mediators, ECM composition, basement membrane formation and matrix stiffness, a combination of which are likely responsible for the differential epithelial phenotype noted in this study. This co-culture platform increases the complexity and relevance of the system further, whilst also providing opportunities to investigate the role of direct and paracrine communication between the two compartments and the influence of tissue-specific fibroblasts on epithelial cell phenotype.

Furthermore, the introduction of CAPE, a well-documented inhibitor of cancer cell migration and proliferation, was found to significantly reduce invasion of the MDA-MB-231 line in both the simple 3D and complex full-thickness models. Although further analysis would be required to determine if this observation is a consequence of reduced cell invasion, proliferation or apoptosis, it supports previous studies that have demonstrated an inhibitory and cytotoxic impact on both the MCF-7 and MDA-MB-231 lines [69,70,71,72,84]. For inducing cell cycle arrest, apoptosis and inhibition of NFκB signalling, CAPE has been proposed as a potential preventative, therapeutic or adjuvant agent, a concept supported by our findings [72]. This proof-of-concept study demonstrates the potential application for these model systems to be utilised as tools capable of elucidation of mechanistic information in relation to candidate compounds. The technology presented in this study is capable of providing fundamental insights into the mechanistic action of therapeutic compounds such as CAPE, including potential roles in ECM turnover and cell–cell junctional complexes that impact stromal–epithelial interactions; however, this is outside the scope of this study. Successful demonstration of CAPE functionality establishes the potential application of these model systems as pre-clinical screening assays, which future studies could build on.

As an initial step in enhancing the biological relevance of existing breast cancer cell lines, this study has demonstrated how increasing the physiological relevance of culture geometry and cell communication can influence epithelial cell phenotype. Future studies would build on these findings by increasing the complexity of the analysis, such as by adding lineage-tracing dyes to stromal–epithelial cultures to aid quantification of invasion and transcriptomic analysis of changes in epithelial cell phenotype at the molecular level. Furthermore, the role of TGFβ supplementation and its impact on fibroblast behaviour would also be further explored. Finally, future studies would benefit from applying this advanced culture technology to patient-derived cells, offering a platform capable of personalised screening. This would also add an element of validation to the system, with which results obtained from established cell lines could be compared.

In this study, we have presented a novel alternative bioengineering approach to recapitulate aspects of the complex architecture of the tumour microenvironment utilising established cancer lineages, further enhancing their value as research tools. This represents a useful advancement in cancer cell modelling, as aspects of stromal–epithelial interactions which are critical to cancer progression are often not represented by existing approaches, which limits their predictive capacity.

## 5. Conclusions

This novel model of breast cancer invasion, combining both mesenchymal and epithelial compartments, offers significant benefits to the study of cancer cell invasion. It allows for fundamental pathogenesis studies at the cell and molecular levels, whilst also lending itself to a myriad of other applications such as drug discovery, pharmacological screening and personalised medicine. Compared with traditional 2D approaches, these systems offer a microenvironment that combines epithelial–stromal communication, physical and biochemical interactions, improved geometry and endogenous tissue-specific ECM, all of which impact cancer disease progression and improve the relevance of widely described breast cancer cell lines. Once this model is benchmarked through application of a broader range of compounds and patient-derived cells, researchers will be able to generate more meaningful data from established breast cancer cell lineages with likely greater predictive value and improved physiological relevance.

## Data Availability

Our supporting research data are published in the Durham University Research Data Repository. DOI: 10.15128/r1gt54kn09q.

## Supplementary Materials

The following supporting information can be downloaded at: https://www.mdpi.com/article/10.3390/cells15201882/s1, Table S1: Antibodies used in immunofluorescence analysis.
