# Supplementary material for: A Novel Bioengineering Approach to Breast Cancer Invasion In Vitro Through Enhancing the Value of Existing Breast Cancer Epithelial Lineages
Source: Cells. 2026 Oct 15;15(20):1882. doi: 10.3390/cells15201882 (PMC13703203; doi:10.3390/cells15201882)
Supplement: Supplementary file 1 [file cells-15-01882-s001.zip › cells-4610644-supplementary.pdf]

Supplementary Table S1: Antibodies used in immunofluorescence analysis

	Target	Supplier	Product Code	Dilution
Primary Antibodies	Fibronectin	Abcam	Ab17808	1:100
	Collagen I	Abcam	Ab34710	1:100
	Cytokeratin 8 (CK8)	Abcam	Ab59400	1:100
	Vimentin	Santa Cruz	Sc6260	1:100
	Collagen IV	Abcam	Ab6586	1:100
	Collagen III	Abcam	Ab7778	1:100
	αsmooth muscle actin (α SMA)	Abcam	Ab5694	1:100
